# Complete mitochondrial genome of *Tringa erythropus* (Charadriiformes: Scolopacidae)

**DOI:** 10.1080/23802359.2016.1219637

**Published:** 2016-09-03

**Authors:** Yuanyuan Cheng, Lizhi Zhou, Yuanqiu Dong

**Affiliations:** a School of Resources and Environmental Engineering, Anhui University, Hefei, China;; b Anhui Biodiversity Information Center, Hefei, China

**Keywords:** Spotted redshank, *Tringa erythropus*, complete mitochondrial genome, gene arrangement

## Abstract

The spotted redshank *Tringa erythropus* is a shorebird in the large bird family Scolopacidae. In this study, we sequenced and characterized its complete mitochondrial genome. The mitogenome is a circular DNA molecule of 16,683 bp in length, containing 13 protein-coding genes, 22 tRNA, 2 rRNA genes, and an AT-rich region. Its gene arrangement pattern is identical with typical bird species. Based on our data combined with the mitgenome DNA sequences of 12 Scolopacidae birds from GenBank, phylogenetic analysis indicated that *T. erythropus*, *Arenaria interpres*, and *Eurynorhynchus pygmeus* formed a group.

Spotted redshank *Tringa erythropus* is a Eurasian wader (shorebird) in the large family Scolopacidaea, which breeds across northern Scandinavia and northern Asia, migrates south to the Mediterranean, the southern British Isles, France, tropical Africa, and tropical Asia for the winter (Dickinson 2003). In this study, we sequenced the complete mitochondrial genome (mitogenome) of the *T. erythropus* with the hope of providing molecular information for further studies of the phylogenetics of Scolopacidae birds. Total genomic DNA was extracted from the muscle tissue using the standard phenol–chloroform protocol, as described by Sambrook and Russell (2001), and the complete mtDNA was amplified and sequenced using 28 primer pairs by referring to close species and used the PCR-based method to obtain the complete mtDNA (Ge et al. 2016; Yu et al. 2016). The tissue was sampled from a dead individual from Hefei Xinqiao International Airport, Anhui Province, China on 7 May 2013. The specimen was kept in the Institute of Biodiversity and Wetland Ecology, Anhui University, China (Voucher TE20130507).

The mitochondrial genome of *T. erythropus* is a circular DNA molecule of 16,683 bp in length (GenBank accession no. KX230491), comprising 13 protein-coding genes, 22 tRNA, 2 rRNA genes, and an AT-rich region. Its gene arrangement pattern is identical with typical bird species (Dong et al. 2016).

The overall base composition is A, 31.56%; C, 30.08%; G, 13.41%; and T, 24.95%. A + T content is 56.51%, higher than C + G content (43.49%). The mitogenome comprises a control region, the gene order is identical to most of the other Charadriiforme birds (Han et al. 2016). All protein-coding genes use the standard mitochondrial initiation codon ATG, except for COI start with GTG. TAN is the most frequent stop codon, and AGN and T- are also occurred very common. The mtDNA sequence contains 985-bp 12S rRNA and 16S rRNA of 1600 bp, which was located between tRNA^Phe^ and tRNA^Leu^, and separated by tRNA^Val^. All tRNAs possess the classic clover leaf secondary structure except for tRNA^Ser (AGN)^ and tRNA^Lys (CUN)^, which lack the ‘DHU’ stem, only forming a simple loop, as observed in other avian mitogenomes (Bernt et al. 2013). The non-coding regions include a control region (D-loop) and a few intergenic spacers. The control region is located between tRNA^Glu^ and tRNA^Phe^, and is 1187 bp in length.

The complete mitogenome sequences of *T. erythropus* combined with other 12 mitogenome DNA sequences from Charadriiformes were used for phylogenetic analysis, setting *Gallus gallus* as an outgroup. Maximum-likelihood method (ML) was used to examine the phylogenetic position of *T. erythropu*s. It appeared that *T. erythropus*, *Arenaria interpres*, and *Eurynorhynchus pygmeus* formed a group (Figure 1). This mitogenome provide an important basic data for systematic analysis of genus *Tringa* and family Scolopacidae.

**Figure 1.**
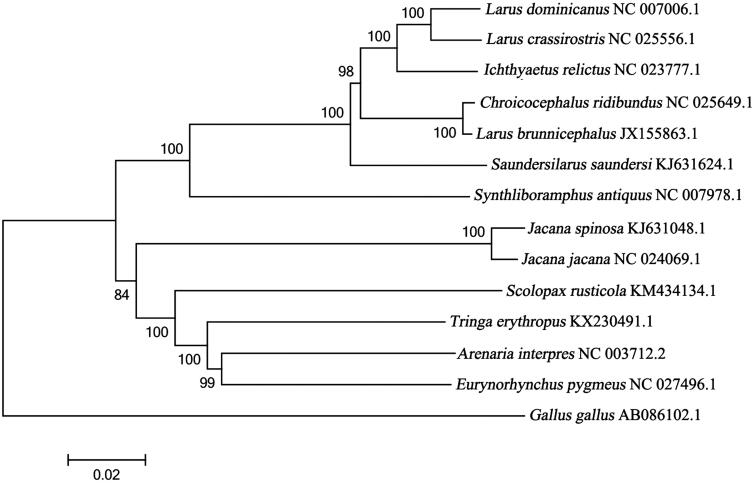
The phylogenetic position of *Tringa erythropus* in some of the Charadriiforme birds. The phylogenetic analysis was conducted using the neighbour-joining method based on 13 complete mtDNA sequences, and *G. gallus* (NC 001323) was used as an outgroup. The phylogenetic tree was built using the Kimura-two-parameter (K2P) model, and the numbers on the branches are bootstrap values. The 13 species Charadriiforme included six Laridae birds: *Larus dominicanus* (NC 007006.1), *L. crassirostris* (NC 025556.1), *Ichthyaetus relictus* (NC 023777.1), *Chroicocephalus ridibundus* (NC 025649.1), *L. brunnicephalus* (JX 155863.1), *Saundersilarus saundersi* (KJ631624.1) and one Alcidae bird: *Synthliboramphus antiquus* (NC 007978.1), two Charadriidae birds: *Jacana spinosa* (KJ 631048.1) and *J. jacana* (NC 024069.1), four Scolopacidae birds: *Scolopax rusticola* (KM 434134.1), *Arenaria interpres* (NC 003712.2), *Eurynorhynchus pygmeus* (NC 027496.1) and *T. erythropus*.
